# Fungal Endophthalmitis: Clinical Characteristics, Pathogens, and Factors Affecting Visual Outcome

**DOI:** 10.3390/antibiotics13030199

**Published:** 2024-02-20

**Authors:** Xiaoxia Li, Zhi Chen, Xiuwen Zhang, Zimei Zhou, Maureen Boost, Taomin Huang, Xingtao Zhou

**Affiliations:** 1 Department of Pharmacy, Eye & ENT Hospital, Fudan University, Shanghai 200031, China; xiaoxia.li@fdeent.org (X.L.); xiuwen.zhang@fdeent.org (X.Z.); 2 Eye Institute and Department of Ophthalmology, Eye & ENT Hospital, Fudan University, Shanghai 200031, China; peter459@aliyun.com; 3 NHC Key Laboratory of Myopia, Fudan University, Shanghai 200031, China; 4 Key Laboratory of Myopia, Chinese Academy of Medical Sciences, Shanghai 200031, China; 5 Shanghai Key Laboratory of Visual Impairment and Restoration, Shanghai 200031, China; 6 Department of Ophthalmology, BronxCare Health System, Bronx, NY 10456, USA; zzhou@bronxcare.org; 7 School of Optometry, The Hong Kong Polytechnic University, Hong Kong SAR 999077, China; maureen.boost@polyu.edu.hk

**Keywords:** *Fungal endophthalmitis*, corneal infiltrate, *Candida* species, *Aspergillus* species, voriconazole, pars plana vitrectomy, visual outcome

## Abstract

Aims: The aims of this study are to investigate the etiology, microbiological spectrum, and risk factors associated with visual outcomes of fungal endophthalmitis (FE) in a tertiary eye specialty hospital in Shanghai, China. Methods: This was a retrospective, single-center case series. The clinical characteristics, etiology, microbiological spectrum, and management, as well as the visual outcomes, were analyzed. Logistic regression was used to analyze the factors related to visual outcomes. Results: This study involved 102 eyes of 92 patients with FE, including 63 males (66.3%). The mean age was 44.4 ± 19.8 years. The most common etiology of FE was trauma (56.5%). The predominant fungal species isolated were *Aspergillus* spp. (31/93, 33.3%). Pars plana vitrectomy (PPV) and intravitreal antifungal agents was performed initially in 86 (84.3%) and 83 (81.4%) eyes, respectively. Only 35 (34.3%) eyes achieved final best corrected visual acuity (BCVA) of 20/400 or better. Ten (9.8%) eyes had a final BCVA of light perception or worse, and five (4.9%) had to be enucleated. The factors determining better visual outcomes included initial visual acuity better than finger-counting (FC) (odds ratio (OR) 5.811, *p* = 0.036), the absence of corneal infiltrate (OR 10.131, *p* = 0.002), and Candida species infection (OR 6.325, *p* = 0.011). Conclusions: Early diagnosis of FE and a timely vitrectomy, combined with an intravitreal injection of an antifungal drug, can mitigate the devastating results of intraocular fungal infection. Not being infected by *Aspergillus* spp., an initial BCVA that was no worse than FC, and the absence of corneal involvement were related to better visual prognosis.

## 1. Introduction

Fungal endophthalmitis (FE) is an inflammatory response caused by fungal infection in the ocular contents and adjacent tissues. It has the characteristics of insidious onset and long incubation period [1,2]. FE is divided into exogenous and endogenous. The main pathogens of exogenous FE were *Aspergillus* and *Fusarium*, while the main pathogens of endogenous FE were *Candida albicans* and *Aspergillus* [3,4]. In the early stage, it is often misdiagnosed or missed due to mild and atypical symptoms [5,6]. FE causes serious damage to intraocular tissues and visual function, and the prognosis of vision is poor [1,7]. Treatment of FE includes medication (antifungal [8], anti-inflammatory [9]) and surgery (removal of intraocular foreign bodies, vitrectomy, silicone oil filling, etc.) [1,2].

Polymerase chain reaction (PCR) detection of 18S/28S ribosomal DNA sequences of fungi in intraocular fluid also helps in the diagnosis [10,11], and can even obtain positive results in some cases with negative culture results, thus increasing the sensitivity of pathogen detection [12]. However, the application and promotion of this technology are also limited by experimental equipment. FE remains a severe intraocular infection that, in most cases, causes vision loss.

Currently, there is no Level One evidence to guide FE management [1], and published data are limited in their spectrum of fungal species and disease management, particularly in China [13,14,15]. However, due to its overlapping clinical signs and symptoms with other endophthalmitis patterns and time-consuming etiological diagnosis, FE poses a challenge for its treatment [16,17]. This study aimed to understand the clinical characteristics of FE over a decade at a tertiary referral center in Shanghai, China, to help clinicians understand changes in the clinical presentation and disease management of FE cases. During this 10-year period, clinical features, initial visual acuity, visual outcome, pathogenic microorganisms, and treatment options were studied in all cases diagnosed with FE.

## 2. Results

### 2.1. Clinical Characteristics

The study included 102 eyes of 92 patients with FE, including 61 males (66.3%) and 31 females (33.7%). The mean age was 44.4 ± 19.8 years (range, 2 to 77 years). The baseline demographic features of the cases are summarized in Table 1. The most common etiology of FE was trauma, accounting for 52 (56.5%) of the 92 cases. An endogenous source was suspected in thirteen (14.1%) cases, followed by postoperative infection in seven (7.6%) cases. Endogenous seeding was mainly attributable to laser fragmentation of urethral stones (*n* = 4, 30.8%), followed by intravenous drug use (*n* = 2, 15.4%). Although ANOVA revealed a significant difference between affected eyes in different sources of infection, this may be attributable to small sample sizes for some etiologies. Cataract was diagnosed in 64 (69.6%) patients, and corneal infiltration was found in 60 (58.8%) patients. Patients with posttraumatic endophthalmitis and postoperative endophthalmitis have more corneal infiltration. The intraocular pressure of 36 (35.3%) eyes was abnormal. Patients with FE developed symptoms an average of seventeen days (range 1 to 179 days) after impact, and the average duration of hospitalization was six days, with patients suffering from postoperative infections having the longest duration of hospitalization.

### 2.2. Microbiology Results

A total of 93 fungi isolates were obtained from 92 patients. Samples from 90 (97.8%) of these 92 patients were culture positive, and 28 (30.4%) microscopy results were positive for fungal species. Vitreous sample results were positive in 68 (66.7%), and aqueous culture results were positive in 18 (17.6%) patients (Table 1). *Aspergillus* spp. (41.3%) were the most common infecting species, followed by *Candida* spp. (30.4%) (Table 2). The predominant fungal species isolated in post-trauma FE were *Aspergillus* spp. (31, 33.3%), and *Candida* spp. (9, 9.7%) was predominant in endogenous FE. Identification at the species level revealed that *C. albicans* was the predominant yeast-causing infection. Although *Aspergillus* spp. were the most common mold species, with only a few isolates of *Fusarium* spp. The majority of isolates were not identified to species level, so the predominant species could not be determined.

### 2.3. Treatment and Outcomes

A total of 102 eyes received 109 PPV. PPV, rather than biopsy, was performed initially in 86 (84.3%) eyes. There was no significant difference in the timing of PPV between causes of FE. Only 10 (9.8%) eyes were treated with vitreous biopsy and antimicrobial injections. Six (5.9%) eyes were initially treated with intravitreal injections of antimicrobials, eventually undergoing PPV with repeated injections of intravitreal antimicrobials because of persistent progressive endophthalmitis. In the patients’ eyes that received antifungal injections, 38, 43, and 2 eyes received intravitreal injection of voriconazole, Amp B, or both, respectively, for initial treatment. The injections were repeated every 48 h for Amp B and every 24 h for voriconazole, as determined by the treating physicians. The median time interval from the commencement of clinical symptoms to intravitreal injection of antifungal drugs was 20 days (range 0–187 days), with trauma patients having the shortest time interval. A total of 64 (69.6%) patients received systemic antifungal drug treatment, and 61 (59.8%) eyes received topical antifungal treatment, the latter most frequently prescribed for trauma patients. A total of 59 (57.8%) patients received intravitreal injection of steroids, and 69 (67.6%) eyes received topical steroids. A vitrectomy after initial tap or vitreous lavage after initial vitrectomy was considered if the initial treatment was rendered noneffective. Overall, 102 eyes received 296 intravitreal injections (105 antibacterial drugs and 191 antifungal drugs), which amounted to an average of 2.9 (range, 1 to 18) intravitreal injection for each eye (Table 3).

### 2.4. Visual Outcome

The median follow-up time was 22 months (range: 3 months to 8 years). The initial BCVA was ≥ 20/400 in seven (7.0%) patients. The final BCVA could not be obtained in 12 (13.04%) patients because of loss to follow-up. Of the 102 eyes, only 35 (34.31%) eyes achieved final BCVA of 20/400 or better. Ten (9.80%) eyes had a final BCVA of no more than light perception, and five (4.90%) eyes were enucleated or eviscerated due to uncontrollable endophthalmitis and pan-ophthalmitis that did not respond to treatment or presented a risk of further spread of inflammation. The distribution of the initial and final BCVA is presented in Table 4. Adverse outcomes at the final follow-up included vascularized corneal scar (four eyes), recurrent retinal detachment (eighteen eyes), epiretinal membrane (four eyes), and optic atrophy (five eyes).

The factors significantly determining better visual outcomes included: initial vision greater than or equal to finger-counting (FC) (odds ratio (OR) 5.811, *p* = 0.036), the absence of corneal infiltrate (OR 10.131, *p* = 0.002), and *Candida species* infection (OR 6.325, *p* = 0.011). (Table 5 and Table S1). There was no significant difference in vision prognosis between the patients’ eyes receiving intravitreal voriconazole and intravitreal Amp B (15/32, 46.9% vs. 13/43, 30.2%, respectively, *p* = 0.143), and marked retinal toxicity was not observed with either intravitreal agent. Age, sex, microscopy positive, the presence of cataracts, combined bacterial infection, and systemic antifungal drugs did not show any correlation to the final visual acuity.

## 3. Discussion

The current study describes clinically and microbiologically diagnosed FE cases over 10 years at a tertiary referral center for ocular emergencies. The current study results indicated that the presence of *Aspergillus*, corneal involvement, initial vision < CF, and PPV > 24 h were the four main factors associated with a poorer visual prognosis.

In 92 patients with fungal endophthalmitis, the proportion of exogenous infection was higher (64.1%), and the proportion of endogenous infection was 14.1%, suggesting that exogenous infection was the main cause of fungal endophthalmitis, which was consistent with the results of other studies [18]. *Aspergillus* (41.3%) was the most common pathogen in FE. In a series of all patients treated for exogenous fungal endophthalmitis at an eye hospital in Florida over a 16-year period, *Candida* spp. accounted for only six of the forty-one cases (15%), whereas mold was found in thirty-five cases (85%) [19], which was consistent with the current study.

Compared to *Candida* endophthalmitis, *Aspergillus*-mediated endophthalmitis is usually more severe, with more extensive areas of retinal necrosis and bleeding. Rao et al. [20] studied the clinical and histopathological features of 25 patients who underwent enucleation for endogenous FE, and found that *Aspergillus* invaded the retinal and choroidal vascular walls, while *Candida* only invaded the vitreous body. The current study found that, consistent with the previous study [21], FE patients infected by *Aspergillus* had significantly poorer visual outcomes compared with patients infected by *Candida.*

Traditionally, a vitreous aspirate has been believed to be more effective than an anterior chamber aspirate for diagnosing bacterial endophthalmitis [19,22], which was consistent with the results of the current study. Importantly, however, seven cases with positive anterior chamber aspirate results had negative vitreous culture results. This may be related to the primary location of the infection. Therefore, both anterior chamber and vitreous aspirates should be performed for a complete endophthalmitis evaluation whenever possible.

An intact corneal epithelium is important in preventing the invasion of various pathogens. Should the corneal epithelium be damaged in the case of trauma or infection, the corneal stroma is rendered unprotected, which can easily lead to oedema and even haze. The combination of these factors adds to the visual loss in FE cases. In this retrospective study, the presence of corneal infiltrate, which was noted in 54 (58.70%) eyes, was significantly associated with a poorer visual outcome, which was consistent with the findings of other studies [16,23].

The prognosis for FE is usually poor due to the difficulty of early diagnosis and the severe damage to the patients’ eye caused by fungal infections. Because the incidence of bacterial infections after penetrating ocular trauma is much higher than that of fungal infections, clinicians tend to treat posttraumatic endophthalmitis with antimicrobial drugs first. As a result, the treatment of FE may be delayed in routine clinical practice [5,6,7]. This study was conducted to investigate the effect of antifungal drugs on visual prognosis during the first vitrectomy, but no significant difference was observed between drug treatment groups. However, it is noteworthy that early vitrectomy (PPV ≤ 24 h) was associated with better visual outcomes (*p* = 0.038). Other studies have also reported the benefits of early vitrectomy in FE [24,25]. The early diagnosis of FE and a timely vitrectomy, combined with the intravitreal injection of antifungal drugs, have been shown to mitigate the devastating outcome of fungal infections in patients’ eyes [26].

The impact of the initial visual acuity on the prognosis of visual acuity was also analyzed, and it was found that the initial visual acuity was a significant predictor: when initial visual acuity was greater than FC, a better BCVA was observed at the last follow-up visit. In the current study, 35 (34.31%) eyes achieved a visual acuity of 20/400 or better. Similar outcomes were reported by Zhuang et al. [27] in a cohort of patients from eastern China diagnosed with posttraumatic FE, with 5.7% of eyes finally enucleated, and 34.3% of eyes achieving a visual acuity ≥ 20/400.

A limitation of the current study was its retrospective nature, which may have led to bias regarding data collection, with substantial individual variability of follow-up and treatment regimen. Polymerase chain reaction, which would have facilitated the earlier detection of fungal infections, was not used for patients in the current study. In addition, the relatively small sample size may not have allowed for an adequate analysis of the effects of voriconazole and Amp B on clinical outcomes. The results indicated that the use of systemic antifungal drugs had no effect on visual outcome. It should be noted that 57.6% of patients in our center received itraconazole for the systemic treatment of FE, which has poor penetration into the vitreous chamber. Oral administration of voriconazole can achieve vitreous levels of nearly 40 percent of plasma levels [28] and, therefore, should have been used in FE cases.

## 4. Materials and Methods

### 4.1. Study Population

This study is a retrospective, noncomparative case series. In this study, the electronic medical records of all patients diagnosed with FE with proven fungal growth on microbiology analysis during the period of 1 January 2012 to 31 June 2022, were reviewed. Those with features of fungal infection seen on clinical ophthalmic examination and suspected cases who were treated for fungal infection without a positive fungal culture result were excluded. The diagnosis of FE was based on patients’ medical histories, as well as an initial ophthalmic examination and a supplementary examination. Information was extracted from both inpatient and outpatient records, including age and gender, etiology, initial treatment regimen, details of treatment, intravitreal and/or systemic antifungal therapy, early (<24 h) or delayed (>24 h) vitrectomy, further surgical procedures, clinical symptoms and signs, vitreous and aqueous humor culture results, follow-up treatment upon discharge, and best corrected visual acuity (BCVA) at the time of primary hospitalization and last follow-up visit.

### 4.2. Pars Plana Vitrectomy

Early pars plana vitrectomy (PPV) was required in cases with the presence of an intraocular foreign body (IOFB), an anterior penetrating injury combined with a traumatic cataract, traumatic vitreous hemorrhage, dead tissue and inflammatory substances, traumatic retinal detachment, or displacement of the crystalline lens. Depending on the presentation, other surgical procedures were combined when appropriate.

### 4.3. Treatment

Intravitreal antibiotic injections were given to most patients as soon as the diagnosis was established. The medication consisted of intravitreal antibiotics (norvancomycin 1 mg/0.1 mL + ceftazidime 2.25 mg/0.1 mL) with or without dexamethasone (0.4 mg/0.1 mL)/triamcinolone (1 mg/0.1 mL)/methylprednisolone (40 mg/0.1 mL). The treatment also included intensive topical antibiotics (ofloxacin 0.3% tid + tobramycin 0.3% tid + gatifloxacin 0.3% tid and prednisolone acetate 1% tid or flurometholone 0.1% tid and the intravenous injection of ceftazidime (1.5 g bid for 7–10 days). Fungal infection was diagnosed with a microscopic or cultural-positive examination of aqueous humor or vitreous samples. FE was treated with an intravitreal injection of voriconazole (100 μg/0.1 mL) or amphotericin B (5 μg/0.1 mL) in combination with oral voriconazole (200 mg, qd) or itraconazole (200 mg bid). Intensive topical antibiotics (fluconazole 0.2% tid, voriconazole 0.5% q2h, or amphotericin 0.5% qid) were administered if they were deemed appropriate by the physician. Additional procedures, including repeated intravitreal antifungals or vitreous surgery (vitrectomy/lavage), depended on the response to treatment and were at the discretion of the physicians.

### 4.4. Specimen Collection and Culture

Undiluted vitreous and aqueous samples were obtained, either via needle aspiration or PPV at the beginning of the surgery, and via injection in all cases, and all specimens (vitreous body, aqueous humor, cornea, secretions, intraocular foreign bodies, etc.) were sent to the Microbiology Department of the Fudan University Eye and ENT Hospital for a direct smear and culture. After staining with 10% KOH solution, calcium fluorescent white, gossypol blue lactate, etc., direct microscopic examination was performed. For the isolation of fungi, the specimens were inoculated on Sabouraud’s dextrose agar (SDA) and incubated at 28 °C and 35 °C for 5–7 days. The fungal isolates grown were initially identified to classify them as mold or yeast based on colony morphology and color.

### 4.5. Statistical Analysis

Descriptive statistics were processed in Microsoft Excel. The statistical analysis was performed with SPSS software (version 22.0; IBM Co., Armonk, NY, USA). Continuous variables were expressed as mean ± standard deviation (SD). According to the standards for the characterization of vision loss updated by the World Health Organization in 2003, the criteria of a favorable vision was set as BCVA ≥ 20/400. The visual acuity of the last visit was used to represent the visual outcome. A favorable functional outcome was defined as an intact retina with BCVA of ≥ 20/400. Pearson’s chi-square tests were used to evaluate clinical characteristics among different groups. The comparison of continuous independent variables with a categorical dependent variable was performed using logistic regression. A value of *p* < 0.05 was considered statistically significant.

## 5. Conclusions

In conclusion, *Aspergillus* is a common etiological agent of FE. Early vitrectomy, combined with intravitreal antifungal therapy, is an effective treatment for FE. Not being infected by *Aspergillus* species, an initial BCVA (logMAR) no worse than FC, the absence of corneal involvement, and timely PPV were factors related to better visual prognosis.

## Figures and Tables

**Table 1:** Clinical characteristics of 92 patients with proven fungal endophthalmitis.

Clinical Characteristics	Total 92/102	Post-Trauma 52/52	Postoperative 7/7	Endogenous 13/16	Other 20/27	*p* Value
Male/Female	61/31	40/12	4/3	7/6	10/10	0.08
Age in years (years) (mean, SD)	44.4 ± 19.8	39.4 ± 20.7	59.1 ± 17.4	44.5 ± 16.1	52.0 ± 16.0	0.16
Infected eyes						
Left eye	42 (45.6%)	19 (36.5%)	6 (85.7%)	8 (61.5%)	9 (45.0%)	<0.001
Right eye	42 (43.5%)	33 (63.5%)	1 (14.3%)	2 (15.4%)	4 (20.0%)	
Both eyes	10 (10.9%)			3 (23.1%)	7 (35.0%)	
Underlying disease						
Diabetes	13(14.1%)	3 (5.8%)	1 (14.3%)	2 (15.4%)	7 (35.0%)	0.018
Hypertension	14 (15.2%)	3 (5.8%)	3 (42.8%)	1 (7.7%)	7 (35.0%)	0.020
Cataract	64 (69.6%)	37 (71.2%)	5 (71.4%)	8 (50.0%)	14 (51.8%)	0.92
Intraocular pressure						
Hyper	12 (11.8%)	6 (11.5%)	2 (28.6%)	2 (12.5%)	2 (7.4%)	0.816
Normal	64 (62.7%)	32 (61.5%)	3 (42.8%)	11 (68.8%)	18 (66.7%)	
Hypo	24 (23.5%)	12 (23.1%)	2 (28.6%)	3 (18.8%)	7 (25.9%)	
No data	2 (2.0%)	2 (3.8%)				
Mixed infections of bacteria and fungi	39 (38.2%)	23 (44.2%)	3 (42.8%)	6 (37.5%)	7 (25.9%)	0.459
Corneal infiltrate	60 (58.8%)	42 (80.8%)	7 (100.0%)	5 (31.2%)	6 (22.2%)	<0.001
Positive samples						
Vitreous humor	68 (66.7%)	34 (65.4%)	4 (57.1%)	11 (68.8%)	19 (70.4%)	0.021
Aqueous humor	18 (17.6%)	11 (21.2%)	4 (21%)	2 (12.5%)	1 (3.7%)	
Cornea	6 (5.9%)	4 (7.7%)	2 (28.6%)			
Secretion	10 (9.8%)	9 (17.3%)	1 (14.3%)			
Intraocular foreign body	3 (2.9%)	3 (5.8%)				
Other	11 (10.8%)	4 (7.7%)	3 (42.8%)	2 (12.5%)	2 (7.4%)	
Mean latent period (days) (median, IQR)	16.5 (4, 48.75)	14 (2.5, 30.5)	60 (17, 61.5)	19 (7, 29.9)	Unknown	0.809
Length of hospitalization (days) (median, IQR)	6 (4, 10)	5 (3, 9)	16 (4.5, 17.5)	3 (1, 5)	3 (1.75, 5)	0.023

IQR, interquartile range; SD, standard deviation.

**Table 2:** Spectrum of fungal isolates from patients with fungal endophthalmitis.

Organism	Total	Post-Trauma	Postoperative	Endogenous	Other
*Fusarium* species	3 (3.3%)	1 (1.1%)			2 (2.2%)
*F. verticillioides*	1 (1.1%)				1 (1.1%)
*F. solani*	1 (1.1%)				1 (1.1%)
Other *Fusarium* spp.	1 (1.1%)	1 (1.1%)			
*Aspergillus* species	38 (41.3%)	31 (33.3%)	4 (4.3%)	2 (2.2%)	1 (1.1%)
*A. niger*	3 (3.3%)	3 (3.2%)			
*A. flavus*	2 (2.2%)	2 (2.2%)			
*A. fumigatus*	1 (1.1%)	1 (1.1%)			
Other *Aspergillus* spp.	32 (34.8%)	25 (26.9%)	4 (4.3%)	2 (2.2%)	1 (1.1%)
*Candida* species	28 (30.4%)	4 (4.3%)	2 (2.2%)	9 (9.7%)	13(14.0%)
*C. albicans*	16 (17.2%)	1 (1.1%)	1 (1.1%)	6 (8.6%)	8 (8.6%)
*C. tropicalis*	1 (1.1%)			1 (1.1%)	
*C. famata*	1 (1.1%)	1 (1.1%)			
*C. parapsilosis*	2 (2.2%)		1 (1.1%)		1 (1.1%)
Other *Candida* spp.	8 (8.6%)	2 (2.2%)		2 (2.2%)	4 (4.3%)
Unidentified species	24 (26.9%)	17 (18.3%)	1 (1.1%)	2 (2.2%)	4 (4.3%)
Total	93	53	7	13	20

Unidentified species: refers to only microscopic detection of fungi, but cannot determine if it is mold or yeast.

**Table 3:** Treatment of fungal endophthalmitis.

Treatment Plan	Total 92/102	Post-Trauma 52/52	Postoperative 7/7	Endogenous 13/16	Other 20/27	*p* Value
Total of pars plana vitrectomy	109	62	6	15	26	0.232
Times of pars plana vitrectomy (mean, SD)	1.1 ± 0.6	1.2 ± 0.7	0.8 ± 0.7	0.9 ± 0.2	1.0 ± 0.3	
Initial vitrectomy instead of biopsy	86 (84.3%)	43 (82.7%)	3 (42.8%)	15 (93.8%)	25 (92.6%)	0.053
Only vitreous biopsy and antimicrobial injections	10 (9.8%)	5 (9.6%)	2 (28.6%)	1 (6.2%)	2 (7.4%)	
First intravitreal injection of antimicrobials						0.001
Voriconazole + antibiotics	8 (7.8%)	5 (9.6%)		2 (12.5%)	1 (3.7%)	
Amp B + antibiotics	13 (12.7%)	11 (21.2%)	1 (14.3%)	1 (6.2%)		
Only antibiotics	19 (18.6%)	16 (30.8%)	1 (14.3%)	1 (6.2%)	1 (3.7%)	
Only voriconazole	30 (29.4%)	9 (17.3%)	2 (28.6%)	6 (37.5%)	13 (48.1%)	
Only Amp B	30 (28.4%)	11 (21.2%)	2 (28.6%)	5 (31.2%)	12 (44.4%)	
Simultaneous intravitreal injection of Amp B and voriconazole	2 (2.0%)		1 (14.3%)	1 (6.2%)		
Time interval from start of clinical symptom to intravitreal injection of antifungal drugs	20 (7, 37.25)	8.5 (3, 20.25)	15 (6.5, 56)	23 (12.5 61.25)	31 (21.5, 51.5)	0.012
Times of intravitreal injection of antifungal drugs (median, IQR)	1 (1, 2)	1 (1, 2)	3 (2.5, 4)	1.2 ± 0.4	1.1 ± 0.3	0.002
Systemic antifungal drugs	64 (69.6%)	35 (67.3%)	6 (85.7%)	8 (61.5%)	15 (75.0%)	0.186
Itraconazole	36 (39.1%)	20 (38.5%)	2 (28.6%)	4 (30.8%)	10 (50.0%)	
Voriconazole	10 (10.9%)	5 (9.6%)	3 (42.8%)		2 (10.0%)	
First use voriconazole, then use itraconazole	18 (19.6%)	10 (19.2%)	1 (14.3%)	4 (30.8%)	3 (15.0%)	
Topical antifungal drugs	61 (59.8%)	42 (80.8%)	6 (85.7%)	6 (37.5%)	7 (25.9%)	<0.001
Topical steroids drugs	69 (67.6%)	35 (67.3%)	2 (28.6%)	11 (68.8%)	21 (77.8%)	0.104
Intravitreal injection of steroids	59 (57.8%)	27 (51.9%)	4 (57.1%)	11 (68.8%)	17 (63.0%)	0.611

Amp B, amphotericin B; IQR, interquartile range; SD, standard deviation.

**Table 4:** Relationship between initial BCVA and final BCVA.

Final BCVA (logMAR)	Initial BCVA (logMAR), *n*							
	NA	NLP	LP	HM	FC	FC-20/400	≥20/400	Total
NA	3	1	6	1	1	0	0	12
Enucleated	0	0	5	0	0	0	0	5
NLP	0	0	3	2	0	0	0	5
LP	0	0	7	3	0	0	0	10
HM	1	0	7	16	3	0	0	27
FC	0	0	3	4	1	0	0	8
≥20/400	1	0	4	12	6	5	7	35
Total	5	1	35	38	11	5	7	102

BCVA, best-corrected visual acuity; NA, not available; NLP, no light perception; LP, light perception; HM, hand motions; FC, finger counting; logMAR, logarithmic minimum angle of resolution.

**Table 5:** Factors affecting visual outcome in fungal endophthalmitis.

Factor	BCVA (logMAR)		
	OR	95% CI	*p* Value
Initial vision ≥ finger counting	5.811	1.126–29.993	0.036
Absence of corneal infiltrate	10.131	2.289–44.834	0.002
Not *Aspergillus* infection	6.325	1.519–26.346	0.011
Trauma	0.255	0.052–1.262	0.094
Normal intraocular pressure	0.763	0.188–3.087	0.704

BCVA, best-corrected visual acuity; OR = odds ratio; logMAR, logarithmic minimum angle of resolution; CI, confidence interval.

## Data Availability

The data presented in this study are available on request from the corresponding author. The data are not publicly available due to privacy or ethical restrictions.

## Supplementary Materials

The following supporting information can be downloaded at: https://www.mdpi.com/article/10.3390/antibiotics13030199/s1, Table S1: Univariate analysis of factors affecting visual outcome in fungal endophthalmitis.
