# Supplementary material for: Fungal Endophthalmitis: Clinical Characteristics, Pathogens, and Factors Affecting Visual Outcome
Source: Antibiotics (Basel). 2024 Feb 20;13(3):199. doi: 10.3390/antibiotics13030199 (PMC10967284; doi:10.3390/antibiotics13030199)
Supplement: Supplementary file 1 [file antibiotics-13-00199-s001.zip › antibiotics-2750312-supplementary.pdf]

Supplementary Materials

Table S1. Univariate analysis of factors affecting visual outcome in fungal endophthalmitis.

**Factor**	**BCVA (logMAR) (≥20/400)**	**BCVA (logMAR) (<20/400)**	***p* Value**
Gender, *n*			
Female	18	18	0.105
Male	17	36	
Age (years), *n*			
<60	3	12	0.092
≥60	32	42	
Cataract, *n*			
Yes	25	36	0.637
No	10	18	
Microscopy positive, *n*			
Yes	12	15	0.515
No	23	39	
Bacterial infection, *n*			
Yes	16	18	0.242
No	19	36	
Intraocular pressure, *n*			
Normal	29	30	0.017
Abnormal	6	22	
Corneal infiltrate, *n*			
Yes	11	44	<0.001
No	24	10	
Trauma, *n*			
Yes	22	20	0.019
No	13	34	
Initial visual acuity, *n*			
≥FC	18	4	<0.001
<FC	16	49	
Aspergillus infection, *n*			
Yes	8	31	0.002
No	27	23	
Intravitreal injection of antibiotics, *n*			
Voriconazole	15	17	0.143
Amp B	13	30	
Systemic antifungal drugs, *n*			
Yes	26	36	0.446
No	9	18	
Systemic antifungal drugs, *n*			

Itraconazole	4	6	0.496
Voriconazole	13	17	
First use voriconazole, then use itraconazole	5	12	
Pars plana vitrectomy, *n*			
≤24 h	33	40	0.038
>24 h	0	7	
Pars plana vitrectomy (≤24 h), *n*			
Intravitreal injection of antifungal drugs	27	29	0.413
Intravitreal injection of antibiotics	6	11	

BCVA, best-corrected visual acuity; logMAR, logarithmic minimum angle of resolution; FC, finger counting; Amp B, amphotericin B.

.
